# Supplementary material for: Chemomechanical regulation of growing tissues from a thermodynamically-consistent framework and its application to tumor spheroid growth
Source: ArXiv. 2024 Dec 3:arXiv:2412.00916v2. Preprint. [Version 2] (PMC11643224)
Supplement: Supplement 1 [file NIHPP2412.00916v2-supplement-1.pdf]

Supplemental Materials for Chemomechanical regulation of growing tissues from a thermodynamically-consistent framework and its application to tumor spheroid growth

A Non-dimensionalization and optimal parameter values

We consider the following non-dimensionalization:

$$\tilde{\mathbf{x}} = \frac{\mathbf{x}}{l}, \quad \tilde{t} = \frac{t}{\tau}, \quad \tilde{\gamma}_c = \gamma_c \tau, \quad \tilde{\gamma} = \gamma \tau, \quad \tilde{\beta} = \beta \tau, \quad \tilde{\alpha} = \alpha \frac{l^2}{\mu \tau}, \; \tilde{\rho} = \frac{\rho}{\rho_0}, \quad \tilde{c} = \frac{c}{c_0}$$

$$\tilde{D} = D \frac{\tau}{l^2}, \quad \tilde{k} = k \frac{\mu}{\rho_0 c_0^2}, \quad \tilde{\eta} = \eta(\mu \tau c_0), \; \tilde{K} = \frac{K}{\mu}, \quad \tilde{c}_H = \frac{c_H}{\mu}, \quad \tilde{W} = \frac{W}{\mu},$$

where l is a characteristic length, τ is a characteristic time, and the tilde quantities are dimensionless. Here we take $l = 1\ \mu m$ and $\tau = 1$ day. The non-dimensionalized system becomes equivalent to the original equations in the main text when we remove all tildes and set $\mu = 1$, and $c_0 = 1$.

**Parameters**	**Incomp. Values**	**Comp. Values**
R_0 : tumor initial radius (μm)	Depends on the experimental data	
K : bulk modulus	-	10
β : elastic relaxation	0	0
η : rescaling factor of the volumetric growth rate γ	0.8	0.7
k : chemical energy constant	2.5	2.5
γ_c : positive constant uptake rate	0.8	1.3
D : diffusional coefficient of nutrient	65^2	90^2
$c_{H,0.7\%}$: shear modulus of 0.7% gel	0.32	0.34
$c_{H,1\%}$: shear modulus of 1% gel	0.82	0.87

Table 1: Values of model parameters in Figure 2A.

B AICc scores

The corrected Akaike information criterion (AICc) [70] is used to determine which model best fits the data. The model with the lower AICc score explains the data better. The AICc score is calculated as

$$\text{AICc} = n\text{Log}(\frac{\text{ERR}}{n} + \frac{2mn}{n-m-1}) \tag{67}$$

where n is the number of observed data points, ERR is the relative error, and m is the number of estimable parameters in the model. The number of data points using for Figure2A and Figure2C is $n = 28$ and $n = 44$, respectively. The AICc predicts that the incompressible model provides a better fit to the data, even though the overall error is smaller for the compressible model. This is because the compressible model contains an additional parameter (the bulk modulus K).

**Parameters**	**Incomp. Values**	**Comp. Values**
R_0 : tumor initial radius (μm)	Depends on the experimental data	
K : bulk modulus	-	30
β : elastic relaxation	0.06	0.08
η : rescaling factor of the volumetric growth rate γ	1.2	1.2
k : chemical energy constant	3.5	3.5
γ_c : positive constant uptake rate	1.2	1.2
D : diffusional coefficient of nutrient	70^2	70^2
$P_{ext,500\text{ Pa}}$: compression pressure for 500 Pa	0.35	0.35
$P_{ext,2K\text{ Pa}}$: compression pressure for 2000 Pa	0.45	0.45
$P_{ext,5K\text{ Pa}}$: compression pressure for 5000 Pa	0.65	0.65

Table 2: Values of model parameters in Figure 2C.

	Gel Confinement		Pressure	
	AICc	ERR	AICc	ERR
Incompressible	**8.9098**	0.8318	**4.1657**	0.4977
Compressible	11.1107	0.7738	5.1822	0.4276

Table 3: AICc scores comparing between incompressible and compressible models

C Frame Invariance of the Dynamic System

We show that the dynamic system of the deformation tensors is frame invariant even with tissue rearrangement. Consider an observer in a frame $\mathbf{x}^+ = \boldsymbol{\chi}^+(\mathbf{X}, t+)$ that connects to the frame $(\mathbf{x}, t)$ via the transformation $\mathbf{x}^+(\mathbf{X}, t+) = \mathbf{Q}(t)\mathbf{x}(\mathbf{X}, t) + \mathbf{c}(t)$, where $\mathbf{Q}(t)$ is an arbitrary orthogonal rotational tensor ($\mathbf{Q}^{\mathrm{T}} = \mathbf{Q}^{-1}$) and $t^+ = t + \alpha$. Without loss of generality, we will assume $\alpha = 0$ and $t^+ = t$. Based on the transformation, we have $\mathbf{F}^+ = \mathbf{QF}$. For the decomposition $\mathbf{F} = \mathbf{F}_e\mathbf{F}_g$, as the translation and rotation only act in the spatial coordinate system, we have $\mathbf{F}_g^+ = \mathbf{F}_g$ but $\mathbf{F}_e^+ = \mathbf{QF}_e$. Given that $(\nabla\mathbf{v})^+ = \mathbf{Q}\nabla\mathbf{v}\mathbf{Q}^{\mathrm{T}} + (d\mathbf{Q}/dt)\mathbf{Q}^{\mathrm{T}}$, we can show that the dynamics of the geometric deformation gradient Eq. (2) is frame invariant:

$$\frac{d\mathbf{F}^+}{dt} - (\nabla\mathbf{v})^+\mathbf{F}^+ = \mathbf{Q}\Big(\frac{d\mathbf{F}}{dt} - (\nabla\mathbf{v})\mathbf{F}\Big) = \mathbf{0}. \tag{68}$$

Since $J^+ = \det(\mathbf{F}^+) = \det(\mathbf{Q})\det(\mathbf{F}) = J$ and $(\nabla\cdot\mathbf{v})^+ = \mathrm{tr}(\mathbf{Q}\nabla\mathbf{v}\mathbf{Q}^{\mathrm{T}}) + \mathrm{tr}((d\mathbf{Q}/dt)\mathbf{Q}^{\mathrm{T}}) = \mathrm{tr}(\nabla\mathbf{v}) = \nabla\cdot\mathbf{v}$ noticing that $(d\mathbf{Q}/dt)\mathbf{Q}^{\mathrm{T}}$ is skew-symmetric, we can show the frame invariance of the dynamics of J Eq. (3)

$$\frac{dJ^+}{dt} = J^+(\nabla\cdot\mathbf{v})^+. \tag{69}$$

Considering the Eulerian growth rate tensor $\mathbf{\Gamma} = \frac{\gamma}{d}\mathbf{I} + \mathbf{\Gamma}_D$ and $\tilde{\mathbf{\Gamma}} = \mathbf{F}_e^{-1}\mathbf{\Gamma}\mathbf{F}_e$, we have $\mathbf{\Gamma}^+ = \mathbf{Q}\mathbf{\Gamma}\mathbf{Q}^{\mathrm{T}}$ and the unaffected $\tilde{\mathbf{\Gamma}}^+ = \tilde{\mathbf{\Gamma}}$. Then we have the frame invariant dynamics of $\mathbf{F}_g^+$

$$\frac{d\mathbf{F}_g^+}{dt} = \tilde{\mathbf{\Gamma}}^+\mathbf{F}_g^+, \tag{70}$$

and the frame invariant dynamics of $\mathbf{F}_e^+$

$$\frac{d\mathbf{F}_e^+}{dt} - (\nabla\mathbf{v})^+\mathbf{F}_e^+ = \mathbf{Q}\Big(\frac{d\mathbf{F}_e}{dt} - \nabla\mathbf{v}\mathbf{F}_e\Big) = -\mathbf{Q}\mathbf{\Gamma}\mathbf{F}_e = -\mathbf{\Gamma}^+\mathbf{F}_e^+. \tag{71}$$

Similarly with the dynamics of J^+, we can show that the dynamics of ρ, J_e and J_g is also frame invariant. Moreover, we can confirm that the evolution of the Finger deformation tensor Eq. (14) is frame invariant:

$$\frac{d\mathbf{B}_e^+}{dt} - (\nabla\mathbf{v})^+\mathbf{B}_e^+ - \mathbf{B}_e^+(\nabla\mathbf{v}^{\mathrm{T}})^+ = \mathbf{Q}\overset{\triangledown}{\mathbf{B}}_e\mathbf{Q}^{\mathrm{T}} = -\frac{2}{d}\gamma\mathbf{B}_e^+ - \big(\mathbf{\Gamma}_D^+\mathbf{B}_e^+ + \mathbf{B}_e^+\mathbf{\Gamma}_D^+\big), \tag{72}$$

where $\mathbf{B}_e^+ = \mathbf{Q}\mathbf{B}_e\mathbf{Q}^{\mathrm{T}}$ and $\mathbf{\Gamma}_D^+ = \mathbf{Q}\mathbf{\Gamma}_D\mathbf{Q}^{\mathrm{T}}$ are the transformed finger deformation tensor and deviatoric Eulerian growth rate tensor respectively, and $\overset{\triangledown}{\mathbf{B}}_e = d\mathbf{B}_e/dt - \nabla\mathbf{v}\mathbf{B}_e - \mathbf{B}_e\nabla\mathbf{v}^{\mathrm{T}}$ is the upper-convective time derivative.

D Viscoelastic behavior of the linearized model

We demonstrate the stress-relaxation behavior in the linearization of our nonlinear model. In the theory of small deformations, we assume that $\mathbf{F} = \mathbf{I} + \nabla \mathbf{u}$, $\mathbf{F}_g = \mathbf{I} + \mathbf{G}$ and $\mathbf{F}_e = \mathbf{I} + \mathbf{E}$, where the displacement $\mathbf{u} = \mathbf{x} - \mathbf{X}$, the growth increment tensor $\mathbf{G}$ and the elastic increment tensor $\mathbf{E}$ are small such that their norms are of order ϵ with $\epsilon \ll 1$. By considering the leading-order approximations, we obtain the linearized stress $\boldsymbol{\sigma} = \boldsymbol{\sigma}_D + \boldsymbol{\sigma}_p$:

$$\boldsymbol{\sigma}_p = K \operatorname{tr}(\mathbf{E})\mathbf{I}, \tag{73}$$

$$\boldsymbol{\sigma}_D = \mu(\mathbf{E} + \mathbf{E}^{\mathrm{T}} - (2/d) \operatorname{tr}(\mathbf{E})\mathbf{I}). \tag{74}$$

To derive the evolution of $\boldsymbol{\sigma}_D$ and $\boldsymbol{\sigma}_p$, we first obtain the linearization of the elastic deformation tensor (12) with $\boldsymbol{\Gamma}_D$ defined in (24):

$$\frac{\partial \mathbf{E}}{\partial t} = \nabla \mathbf{v} - \frac{\gamma}{d}\mathbf{I} - \beta\Big(\mathbf{E} + \mathbf{E}^{\mathrm{T}} - \frac{2}{d} \operatorname{tr}(\mathbf{E})\mathbf{I}\Big), \tag{75}$$

where we have omitted the advection term $\mathbf{v} \cdot \nabla \mathbf{E}$ of smaller order. It yields the evolution of isotropic stress (or pressure)

$$\frac{\partial \boldsymbol{\sigma}_p}{\partial t} = K(\nabla \cdot \mathbf{v} - \gamma)\mathbf{I}, \tag{76}$$

and the stress relaxation behavior of viscoelastic materials for deviatoric stress

$$\boldsymbol{\sigma}_D + \frac{1}{2\beta}\frac{\partial \boldsymbol{\sigma}_D}{\partial t} = \frac{\mu}{2\beta}\Big(\nabla \mathbf{v} + \nabla \mathbf{v}^{\mathrm{T}} - \frac{2}{d}(\nabla \cdot \mathbf{v})\mathbf{I}\Big), \tag{77}$$

where $(\nabla \mathbf{v} + \nabla \mathbf{v}^{\mathrm{T}})$ defines the strain rate, β serves as half the rate of stress relaxation and the ratio $\mu/(2\beta)$ plays the role of "effective" viscosity. This Maxwell-like model of deviatoric stress matches exactly with the linearzation of our previous nonlinear elastic model with "adaptive reference map" [34], where we describe the tissue rearrangement by the adaption of reference state to the current deformed state via $d\mathbf{X}/dt = \tilde{\beta}(\mathbf{x} - \mathbf{X})$ and the two relaxation coefficients are related by $\tilde{\beta} = 2\beta$.

E Finding the Equilibrium Radius

For incompressible tumor, when $\beta = 0$, we found that $\gamma(r) \equiv 0$ in the entire tumor at equilibrium. Based on this observation, we obtain a system that connects the equilibrium radius R and the solution of fe_θ as below

$$P - \frac{1}{fe_\theta^4} = \int_R^r \frac{2}{s}(\frac{1}{fe_\theta^4} - fe_\theta^2)ds + F_{ext}, \tag{78}$$

$$\frac{1}{2}(2fe_\theta^2 + \frac{1}{fe_\theta^4} - 3) + P = \frac{k}{2}c(R,r)^2 + \frac{1}{3}(2fe_\theta^2 + \frac{1}{fe_\theta^4}), \tag{79}$$

where the first equation comes from the force balance equation and the second equation comes from the fact $\gamma(r) \equiv 0$, and $c(R,r)$ is the analytical solution of the quasi-equilibrium reaction diffusion equation (26), namely $L^2 \frac{1}{r^2}\frac{\partial}{\partial r}(r^2 \frac{\partial c}{\partial r}) - \gamma_c c = 0$, given by

$$c(R,r) = \frac{R \sinh(\frac{\sqrt{\gamma_c} r}{L})}{r \sinh(\frac{\sqrt{\gamma_c} R}{L})}, \tag{80}$$

where we use $L = 65$ and $\gamma_c = 0.7$ without loss of generality. The solutions of (78) and (79) satisfy the following boundary conditions:

$$\begin{aligned} fe_\theta &= 1 \text{ at } r = 0, \\ P - \sigma_{rr} &= F_{ext} \text{ at } r = R \\ c &= 1 \text{ at } r = R. \end{aligned}$$

Letting x be fe_θ^2, we substitute P from equation (79) to equation (78) as follows

$$\frac{k}{2}c(R,r)^2 - \frac{1}{2}(2x + \frac{1}{x^2} - 3) - \frac{2}{3}(\frac{1}{x^2} - x) - F_{ext} = \int_R^r \frac{2}{s}(\frac{1}{x^2} - x)ds \tag{81}$$

If we multiply equation (81) by x^2 and rearrange the terms, we obtain a cubic polynomial of x as below

$$H(x,r;R) := (-\frac{1}{3})x^3 + \left(\frac{k}{2}c(R,r)^2 + \frac{3}{2} - F_{ext} - \int_R^r \frac{2}{s}(\frac{1}{x^2} - x)ds\right)x^2 - (\frac{7}{6}) = 0, \tag{82}$$

where the coefficients depend on r and R. In other words, given an equilibrium radius R, we can obtain the solution x at position $r \in [0,R]$ based on the above equation. Based on our numerical calculations, we found that fe_θ monotonically decreases from the center of the tumor to the boundary when $\beta = 0$. Furthermore, since we have $fe_\theta = 1$ at $r = 0$ and $fe_\theta > 0$ for all r based on its physical interpretation, we can conclude that $x \in (0,1]$ for $r \in [0,R]$. We also confirm this result $fe_\theta \in (0,1]$ by numerical simulations.

Now let us consider the equation (82) at $r = 0$

$$\frac{k}{2}c(R,0)^2 - \int_R^0 \frac{2}{s}(\frac{1}{x^2} - x)ds = F_{ext}. \tag{83}$$

If $F_{ext} = 0$, it is not possible to find an equilibrium solution for $0 < R < \infty$ since the left hand side is positive given that $c(R,0) > 0$ and $k > 0$ and $x \in (0,1]$. Furthermore, the radius R will increase indefinitely due to the positive external driving force expressed by the left hand side.

Next, consider the equation (81) at $r = R$, which yields

$$-\Big[\frac{1}{2}(2x + \frac{1}{x^2} - 3) + \frac{2}{3}(\frac{1}{x^2} - x)\Big] = F_{ext} - \frac{k}{2} \tag{84}$$

where we have used the fact $c(R, R) = 1$ at $r = R$. Since $x \in (0, 1]$, the left hand side is non-positive. For a valid solution $x \in (0, 1]$ to exist, we would require that $F_{ext} \leq \frac{k}{2}$. If the constant external pressure is too large, i.e., $P_{ext} > \frac{k}{2}$, there does not exist an equilibrium radius R and the radius R will decrease to zero due to the larger external pressure. However, when gel confinement is present ($c_H > 0$), an equilibrium radius will always exist because F_{ext} depends on R by the relation in Eq. (42). When $F_{ext} > \frac{k}{2}$, the radius R decreases and meanwhile F_{ext} also decreases, and the radius R will reach an equilibrium once F_{ext} is small enough such that the force balance in Eq. (84) holds for some $x \in (0, 1]$.

Assuming an equilibrium radius R exists, we use shooting method to find the appropriate value for R. Specifically, given an initial guess for equilibrium radius R, we can compute $x(r)$ from $r = R$ to $r = 0$ and then update the radius R by matching the boundary condition $x = 1$ at $r = 0$. In particular, we discretize along the radius by $r_i = i\Delta r$ $(i = 0, 1, \dots, N)$ with $\Delta r = R/N$ and use the right endpoint rule to approximate the integral. By considering Eq. (82) with the integral from r_j to R for $j = N, \dots, 0$, we can solve $x(r_j)$ sequentially. In other words, $x(r_j)$ depends on the values of $x(r_i)$ for all $i = j + 1, \dots, N$ that have been obtained before. When solving the value of each $x(r_j)$, if no positive real root exists for Eq. (82), we will assume $x(r_j) = x(r_{j+1})$ and move on to compute $x(r_{j-1})$. While this assumption is not physical, it aids in obtaining numerical values of $x(r_j)$ for all j. If there are multiple real roots for Eq. (82), we will choose the one with the smallest positive value. Notice that, there could be critical numerical errors from approximating the integral when r is small (close to zero) because of the singular factor $\frac{2}{s}$. Instead using the integrand directly, we can approximate $x \approx 1 + \frac{1}{2}x_{rr}(r)r^2$ using Taylor's expansion around $r = 0$ using the condition $x = 1$ at $r = 0$. Then the integral is rewritten as

$$\int_R^r \frac{2}{s}(-\frac{x_{rr}^3}{8}s^6 - \frac{3x_{rr}^2}{4}s^4 - \frac{3x_{rr}}{2}s^2)\frac{1}{(1 + \frac{1}{2}x_{rr}s^2)^2}ds.$$

Since we have used the boundary condition $x(0) = 1$, we eventually match the equation in Eq. (82) rather than the boundary condition. In particular, in order to find the equilibrium tumor radius R, we perform the bisection method with the residual of the equation $H(R)$ in Eq. (82). When $H(R) < 0$, it indicates that the given R is smaller than the equilibrium. Otherwise, choose the other section for new interval until it converges. In our setup, we use this method when r is less than 10^{-3} and the tolerance for the method is 10^{-16}.

F Supplemental Figures

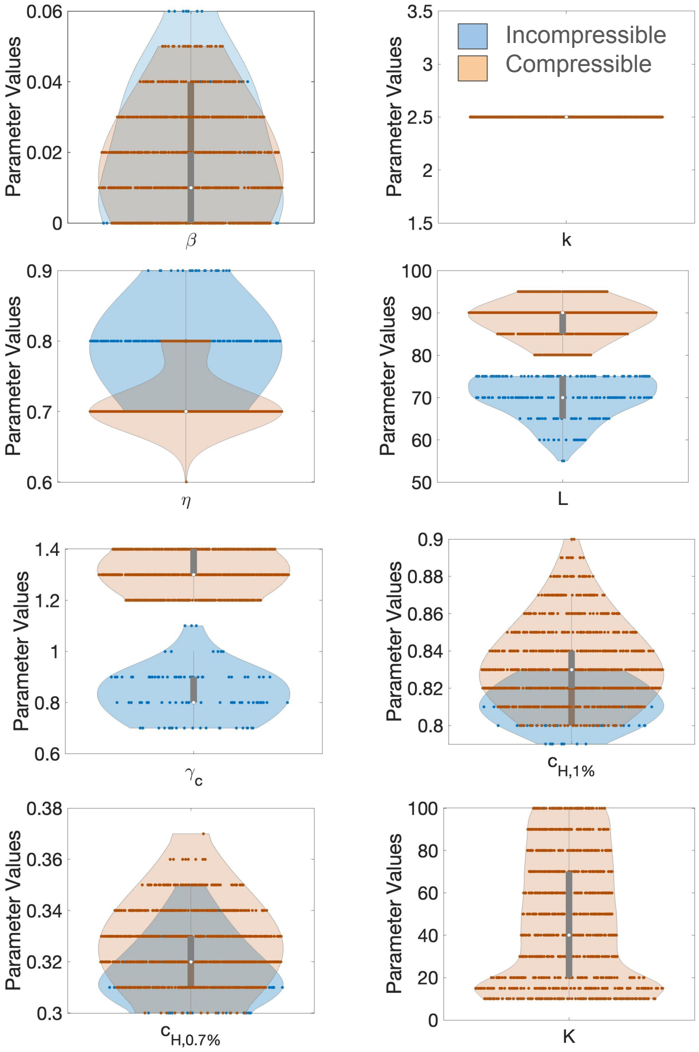

Figure S1: Violin plots displaying the distribution of data sets that are within 10% of the best fitting relative error from Figure 2A. The plots represent each parameter where blue is for the incompressible model and red is for the compressible model. The width of the violin at any given point represents the density of data points at that value. Additionally, there is a central box-and-whisker plot, which provides summary statistics such as the median, quartiles, and outliers of the dataset within each category. Notably, the parameters take distinct values for the compressible and incompressible cases, particularly for $L = \sqrt{D}$ and γ_c. These disparities may rise from complex compensatory effects among other parameters, such as K and c_H.

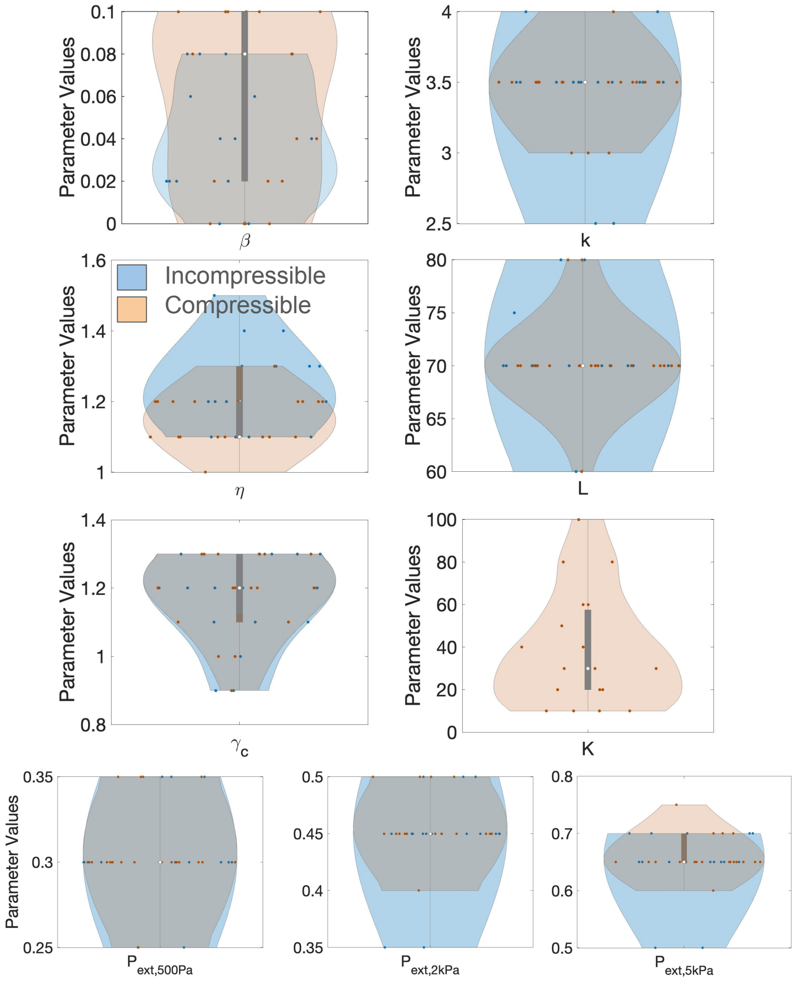

Figure S2: Violin plots showing the distribution of data sets that are within 10% of the best fitting relative error from Figure 2C. The plots present each parameter where blue designates the incompressible model and red designates the compressible model. The width of the violin at any given point represents the density of data points at that value. Additionally, there is a central box-and-whisker plot, which provides summary statistics such as the median, quartiles, and outliers of the dataset within each category. These plots highlight the similarities of the best-fitting parameters between compressible and incompressible models.

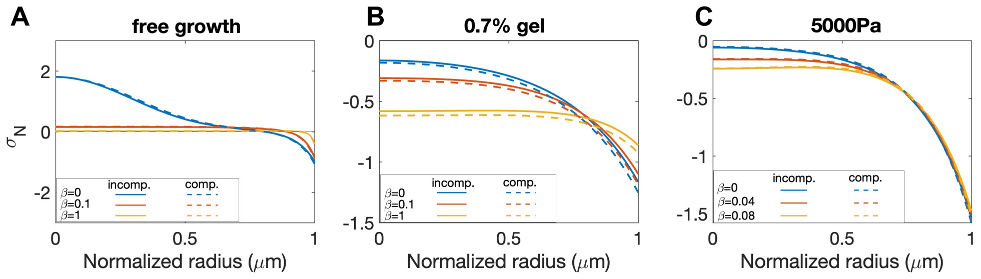

Figure S3: Study of the parameter β where A), B), and C) display the normal stress average σ_N distribution at T=100 for free case, with gel confinement (0.7% gel), and with an applied static external pressure (5000 Pa), respectively.

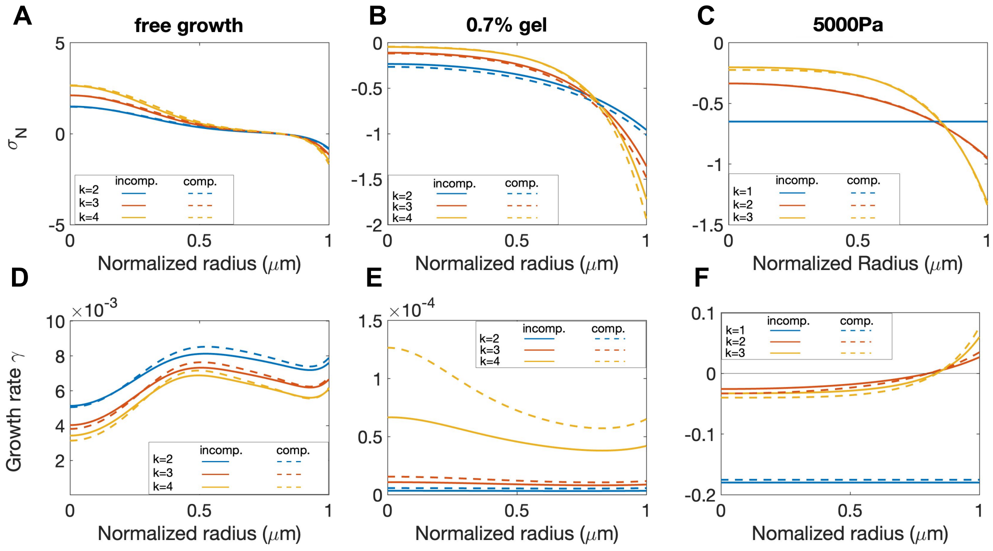

Figure S4: Study of the parameter k where A), B), and C) display the normal stress average σ_N distribution at T=100 for free case, with gel confinement (0.7% gel), and with an applied static external pressure (5000 Pa), respectively. D), E), and F) display the growth rate γ distribution at $T = 100$ for free case, with gel confinement (0.7% gel), and with an applied static external pressure (5000 Pa), respectively.

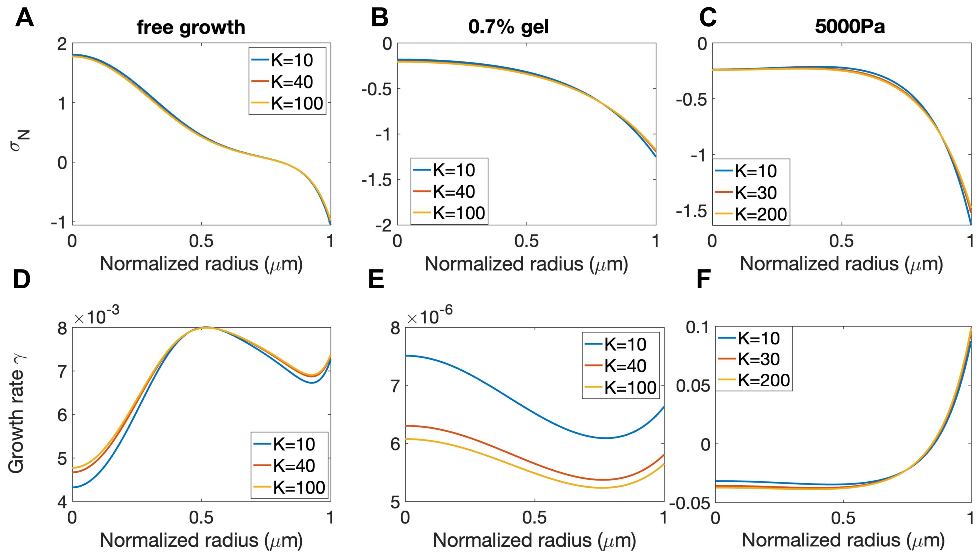

Figure S5: Study of the parameter K where A), B), and C) display the normal stress average σ_N distribution at T=100 for for free case, with gel confinement (0.7% gel), and with an applied static external pressure (5000 Pa), respectively. D), E), and F) display the growth rate γ distribution at $T = 100$for free case, with gel confinement (0.7% gel), and with an applied static external pressure (5000 Pa), respectively.

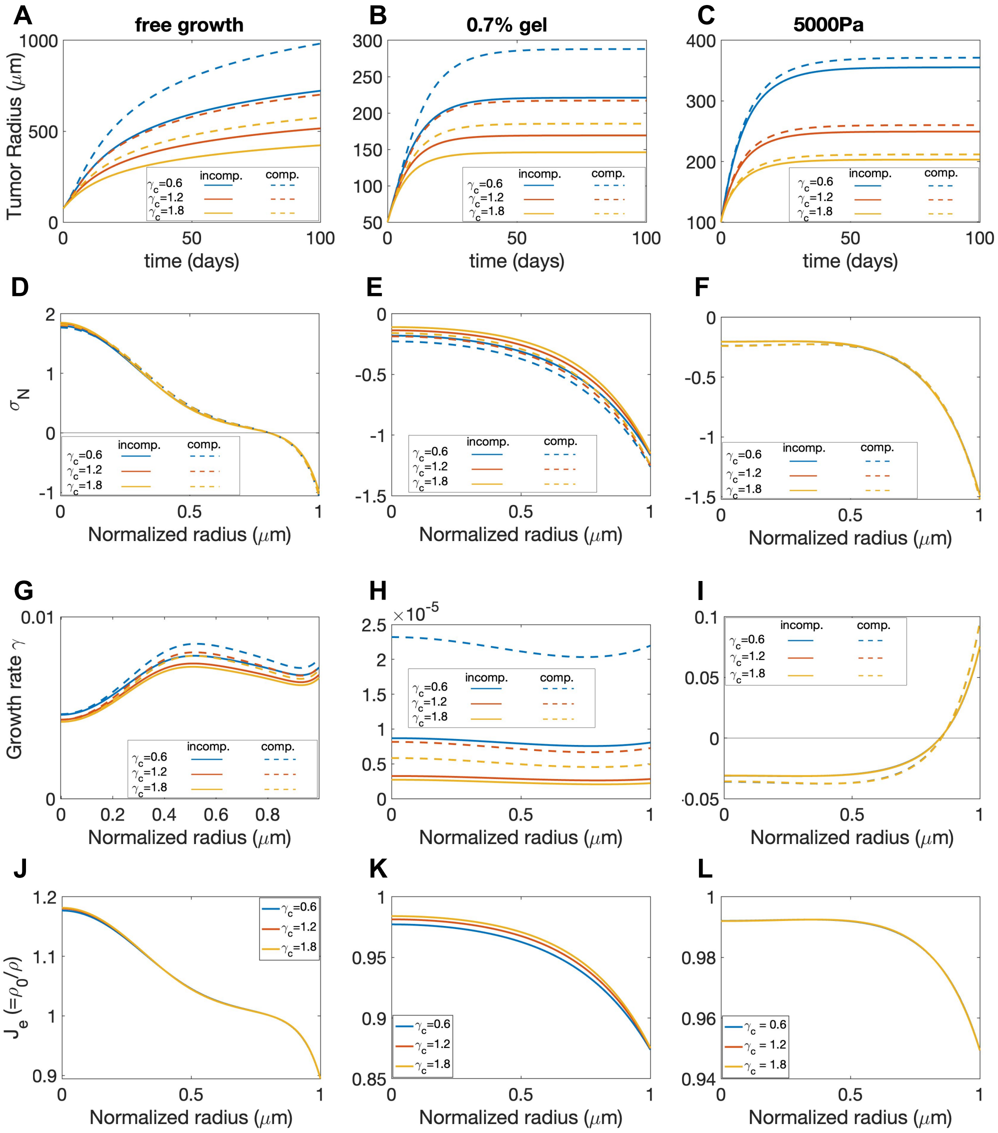

Figure S6: Study of the parameter γ_c, which characterizes the uptake rate in our nutrient transportation equation for free case, with gel confinement (0.7% gel), and with an applied static external pressure (5000 Pa). Figures A) & B) and C) show the evolution of tumor for each case along time (days). Figures D) & E) and F) show the normal stress average σ_N distributions in the normalized radial direction at time $T = 100$. Figures G) & H) and I) indicate the volumetric growth rate distributions in the normalized radial direction at time $T = 100$. The solid lines indicate incompressible results and dashed lines indicate compressible results. Figure J) & K) and L) display the elastic volumetric variation at time $T = 100$ for compressible model.

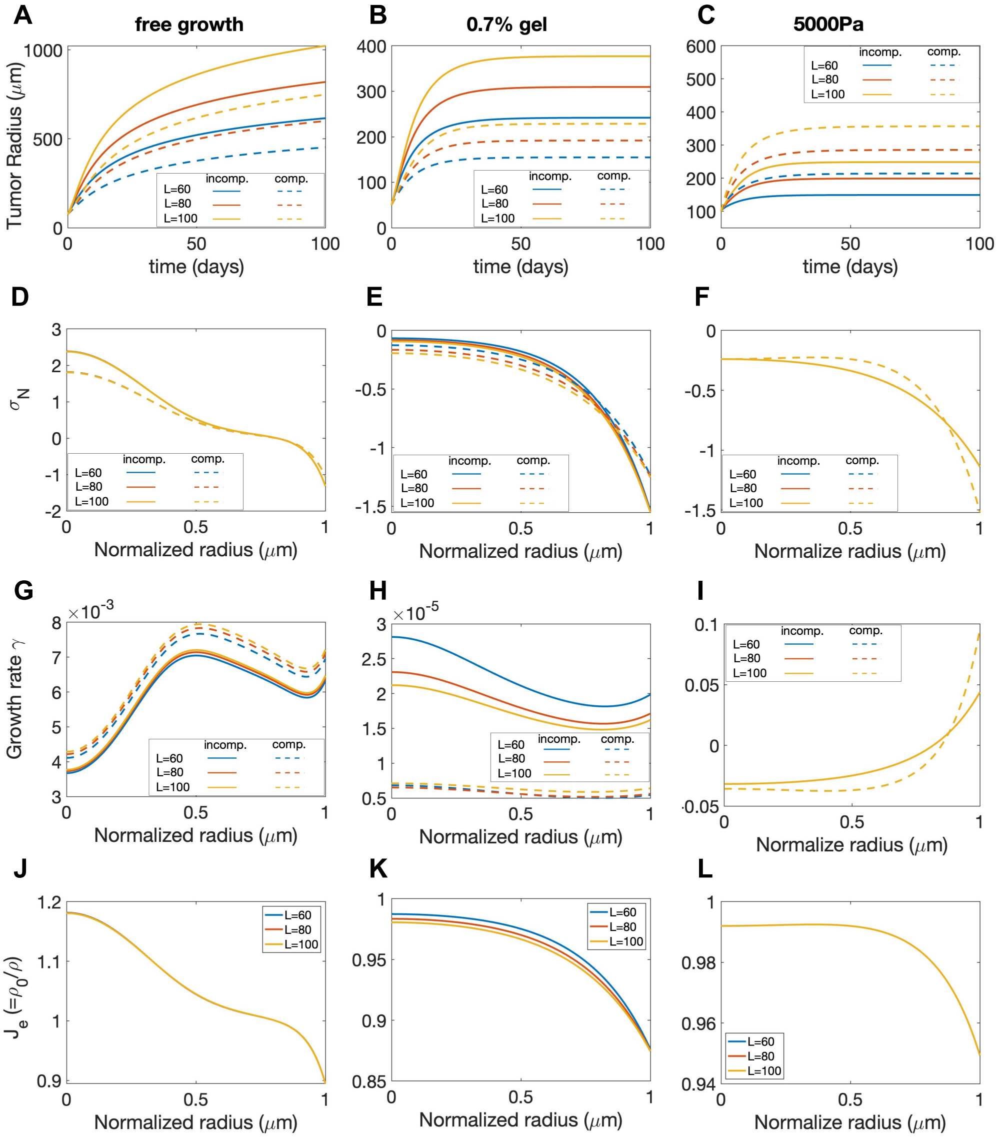

Figure S7: Study of the parameter $L = \sqrt{D}$, which characterizes the diffusion length ($D = L^2$) in our nutrient transportation equation for free case, with gel confinement (0.7% gel), and with an applied static external pressure (5000 Pa). Figures A) & B) and C) show the evolution of tumor for each case as a function of time (days). Figures D) & E) and F) indicate the normal stress average σ_N distributions in the normalized radial direction at time $T = 100$. Figures G) & H) and I) indicate the volumetric growth rate distributions in the normalized radial direction at time $T = 100$. Figures J) & K) and L) show the elastic volumetric variations at time $T = 100$ for the compressible model. The solid lines indicate incompressible results and dashed lines indicate compressible results.
